# Muscle Endurance Training in a Person with Friedreich’s Ataxia

**DOI:** 10.3390/muscles4010001

**Published:** 2025-01-09

**Authors:** Nicole T. McGarrell, Max E. Green, Kevin K. McCully

**Affiliations:** 1 Department of Kinesiology, University of Georgia, Athens, GA 30602, USA; 2 Northside Hospital, Atlanta, GA 30342, USA; 3 InfraredRx, Inc., Athens, GA 30602, USA

**Keywords:** near-infrared spectroscopy, NIRS, neuromuscular electrical stimulation, neuromuscular disease

## Abstract

Friedreich’s ataxia (FRDA) results from a faulty mitochondrial protein known as Frataxin. The purpose of this case report was to test whether skeletal muscle in FRDA can adapt to an endurance-based training program using neuromuscular electrical stimulation (NMES). A 36-year-old female with FRDA completed twelve training sessions, each lasting 30 min over 30 days, focused on the forearm muscles using NMES. Pre- and post-training session measurements of contractions, muscle-specific endurance, and muscle mitochondrial capacity were taken per training session. Training contractions increased from 4200 to 9420. Muscle-specific endurance increased by 14% at 2 Hz and 17% at 4 Hz. Muscle endurance at 6 Hz increased from 0% to 51%. The rate constant of mitochondrial capacity was 0.95 min^−1^ pre- and 0.99 min^−1^ post-training session. In conclusion, one month of NMES increased training volume and muscle-specific endurance but did not change mitochondrial capacity. Muscle adaptations to endurance training were seen in FRDA, but increased training might be needed to test if mitochondrial capacity can improve.

## 1. Introduction

Friedreich’s Ataxia (FRDA) is an autosomal recessive mitochondrial disease that is caused by a mutant Frataxin (FXN) gene [1]. The mutations result in increased numbers of GAA repeats on the shorter allele of the FXN locus. Greater numbers of repeats are associated with a more rapid progression and severity of symptoms [2]. Physical problems such as poor coordination, gait and limb ataxia, and leg weakness are usually present. Although it is a rare disease, FRDA is the most common form of ataxia [3,4,5]. Exercise is recommended for patients with FRDA based on general good practice evidence [6] or moderate scientific evidence [7]. Most research on how people with FRDA respond to exercise has focused on occupational or physical therapy [8]. A case report showed improvements in whole-body exercise capacity with endurance training [9]. However, little is known about how skeletal muscle or skeletal muscle mitochondria in people with FRDA respond to exercise training. Because most studies of FRDA evaluate people who are mobile and can perform voluntary exercise, it is important to extend research studies to people with FRDA who have advanced symptoms and severely reduced mobility.

People with FRDA have been shown to have reduced muscle mitochondrial capacity [10]. Using samples taken from muscle biopsies, FRDA has been shown to be associated with reductions in complex II and IV in particular [11]. While impairments in mitochondrial complexes are informative, it is not clear how the changes in the individual cytochromes from the biopsy data influence overall mitochondrial capacity. It is also not clear if this reduction is a result of reduced FXN levels or the inactivity associated with the disease. Inactivity has been associated with reduced mitochondrial capacity [12]. In addition, it is possible for some mitochondrial deficits to be compensated for with increased mitochondrial number. Thus, there is a need to better understand how in vivo muscle mitochondrial capacity responds to exercise.

The aim of this case report was to evaluate the adaptive response of skeletal muscle in a person with FRDA to endurance-based exercise training. Neuromuscular electrical stimulation (NMES) training was used as it can be applied to people with FRDA who are either mobile or immobile. NMES training has been used successfully to enhance muscle function and mitochondrial capacity in people with motor complete spinal cord injuries [13,14]. This study was based on a similar case report that evaluated the result of a one-month training program on a patient with mitochondrial myopathy [15]. We hypothesized that skeletal muscle in a person with FRDA would positively adapt to a one-month duration stimulation training program, showing increased training levels, increased muscle endurance, and increased muscle mitochondrial capacity (mVO_2_max).

## 2. Results

**Figure 1:**
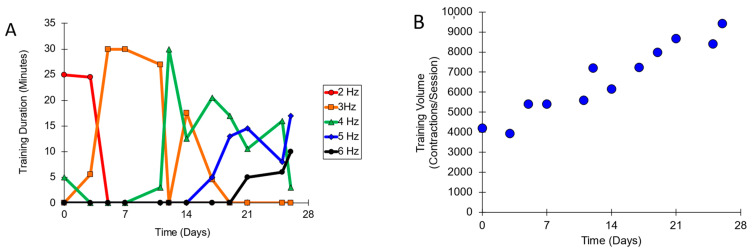
(**A**) The number of minutes at each stimulation frequency over the course of training. (**B**) The total number of muscle contractions at all frequencies during each training session.

The training protocol was well tolerated by the subject, and all training sessions were performed on the scheduled days. Supplementary data are available (FA Summary Graph). The number of contractions per training session increased over the 12 training sessions (Figure 1). The increase in contraction number was related to the use of gradually higher stimulation frequencies. The subject tolerated higher stimulation frequencies and reported increasingly lower pain ratings during stimulation. The pain was assessed using a 10-point scale (0 is no pain, and 10 is the highest imaginable pain level). Over the course of training, there were also fewer requests to turn down the stimulation frequency after it had been turned up.

**Figure 2:**
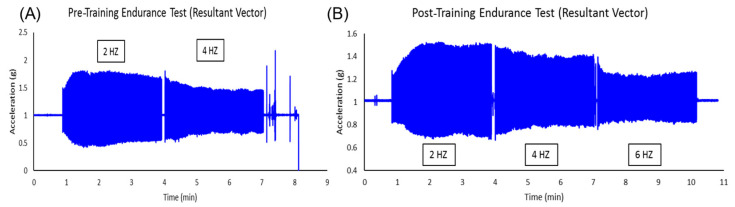
Muscle acceleration during electrical stimulation before training (**A**) and after training (**B**).

**Figure 3:**
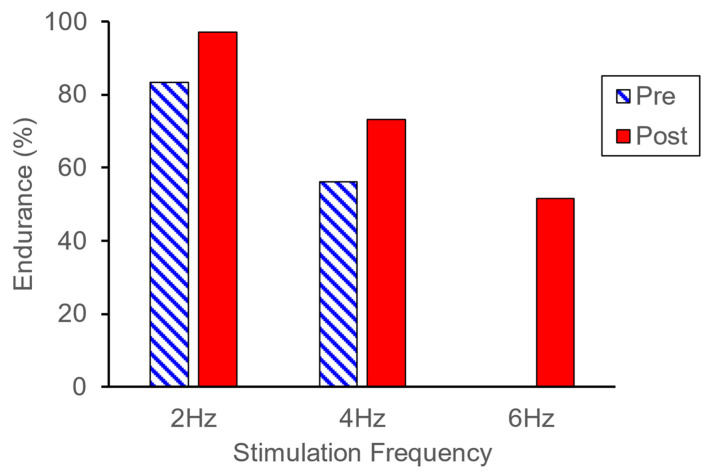
Endurance index values before and after NMES training. Post-training values were higher than the pre-training values at 2 and 4 Hz. The endurance index value was not able to be measured at 6 Hz prior to training.

Endurance test acceleration values before and after NMES training are shown in Figure 2. Prior to NMES training, data from 2 Hz and 4 Hz were collected. Data from 6 Hz were not obtained prior to training because a muscle cramp occurred during stimulation, and stimulation was discontinued. Post-NMES training, data from 2 Hz, 4 Hz, and 6 Hz were collected. The Endurance Index (EI) was higher at 2 Hz and 4 Hz after training compared to before training (Figure 3).

**Figure 4:**
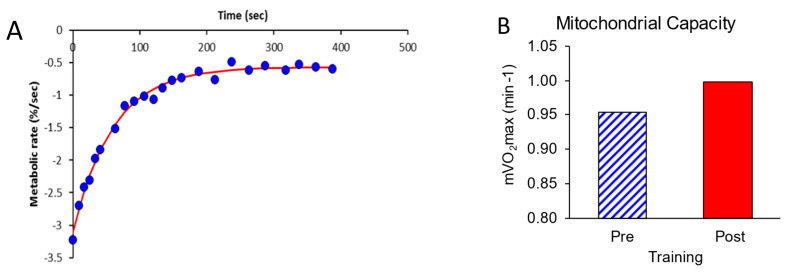
(**A**) Representative recovery curve for measurement of mVO_2_max. The blue circles are individual mVO_2_ measurements. The red line is the exponential fit. (**B**) mVO_2_max values before and after NMES training.

mVO_2_max values were measured before and after NMES training. One of the two pre-training recovery curves is shown in Figure 4A. For muscle activation, the ratio of the first mVO_2_ metabolic rate to the end recovery mVO_2_ rate was 5.4 prior to training and 4.5 after training. These ratios were above the value of 3.0 considered necessary to obtain accurate recovery rate constants. The average mVO_2_ values before and after NMES training are shown in Figure 4B. The rate constant for mitochondrial capacity was 5% higher after NMES training.

## 3. Discussion

The most significant finding of this study was that in a participant with FRDA, muscle function improved after one month of NMES training. This finding was based on more than a doubling of the number of twitch contractions per training session (4200 to 9420). This was similar to the increases in twitch contraction numbers that were previously reported in an NMES training study of the leg muscles of people with motor complete spinal cord injury [16]. In that study, the subject with SCI demonstrated an increase of 1200 to 11,000 contractions in the first month of training. There was also an increase in the endurance index at the three frequencies. Increases in the muscle endurance index were also seen in a case study involving the training of a patient with mitochondrial myopathy [15]. What is remarkable in the current case report is that there are very few exercise training studies involving people with FRDA with which to compare our results. Our results are consistent with a previous case study on FRDA, which showed that 27 voluntary cycling training sessions increased whole-body maximal oxygen consumption by 27% [9]. Taken together, these studies suggest that exercise training can improve muscle function in people with FRDA.

mVO_2_max was 5% greater after NMES training. However, this magnitude of increase was not considered to be great enough to constitute a meaningful improvement. Coefficients of variation for repeated mVO_2_max measurements were shown to be 8–12% [17], greater than the change seen in this study. For comparison, a previous study on able-bodied people has shown that one month of voluntary training of the forearm muscles increased mVO_2_max by 64% [12]. A previous case report with a subject with mitochondrial myopathy using one month of voluntary wrist flexion exercise demonstrated an increase in mVO_2_max by 58% [15]. Four months of a similar twitch-based NMES increased mVO_2_max by 119% in people with spinal cord injuries [16]. It is possible that higher training intensities or more training sessions would have increased mVO_2_max by enough to be considered significant in our participant with FRDA. This conclusion is based on studies involving able-bodied subjects and subjects with spinal cord injuries who used higher exercise intensities and trained for longer periods of time.

Most studies using subjects with FRDA have included people who were ambulatory or could use a recumbent cycle [6,7]. This is typically carried out in order to test whether functional improvements have occurred, such as improvements in walking speed or in power output on a cycle ergometer. In contrast, our study participant lacked voluntary lower leg movement and had very low self-reported physical activity levels. Endurance index values for our participant were lower than reported in a larger study of people with FRDA (50% at 4 Hz versus 40–80% at 6 Hz) [18]. mVO_2_max values were also lower in the current participant than mVO_2_max values reported in a previous study (0.9 min^−1^ compared to 1.1–2.2 min^−1^) [18]. Our participant had lower Barthel index values than most of the subjects in the previous study. The lower endurance index values and mVO_2_max measurements in our participant were consistent with the correlations between these variables and Barthel index values from a previous study [16]. One of the major advantages of NMES training, as well as the measurements of muscle-specific endurance and mVO_2_max, used in this study is that they can be performed by people with a wide range of functional abilities. This includes both people who are ambulatory, and people who are unable to walk or use a recumbent cycle. Using these more inclusive methods opens the possibility of testing a wider phenotypical range of people with FRDA. In addition, the positive muscle adaptations to NMES training may be expected to occur across the wide phenotypical range of people with FRDA [19].

Regular exercise training has been associated with numerous health benefits in able-bodied populations [20]. These benefits include increased muscle mass and muscle endurance leading to increased functional ability. Benefits also extend to improved whole-body metabolism, including better-controlled blood sugar levels and lower cholesterol levels [21,22,23]. Other benefits include reduced risk of cardiovascular disease [24,25]. Overall, increased physical activity has been associated with reduced healthcare costs [26]. Exercise training has been associated with psychological benefits, including reduced anxiety, reduced pain levels, and better sleep quality [27]. NMES training has been associated with health benefits similar to those of voluntary exercise. This is especially true in people who have a reduced ability to perform voluntary exercise [13,14]. Reducing the duration of sedentary time has been shown to have health benefits independent of the total duration of physical activity [28], and short bouts of NMES throughout the day have the potential to reduce the negative effects of long periods of sedentary time. Additional studies are needed to better understand the role of regular exercise on health in people with FRDA and whether regular exercise influences the progression of FRDA symptoms. But it is important to note that the benefits of exercise on health extend beyond increases in functional capacity, and that the benefits of exercise can extend to people with limited functional mobility.

One of the limitations of this study is that it is a single case report. It is possible that other people with FRDA would respond differently to training than our participant. In particular, it was not clear if the inability to perform the 6 Hz portion of the endurance index before NMES training was a unique or consistent response. As pointed out above, very few training studies have been performed with people with FRDA [29]. Additional and larger studies are needed. This study also did not include long-term follow-up to determine if the response to NMES was retained or whether stopping NMES training resulted in a return to the pre-training values. This study also used a person with advanced symptoms related to FRDA. While this demonstrated that people with mobility limitations could perform exercise training with electrical stimulation, future studies are needed to test if such training in people with higher mobility levels also produces meaningful functional gains. In patients who can walk, NMES has been shown to improve gait speed in patients after stroke [30] and in patients with multiple sclerosis [31].

## 4. Materials and Methods

### 4.1. Participant

The participant was a 36-year-old female with Friedreich’s Ataxia. The GAA repeat number was unknown. The GAA repeat number has been associated with greater disease progression [2]. She measured 1.74 m in height and 58 kg in weight, with a BMI of 19.2. This participant was immobile with limited head and arm movement. The participant had a Barthel Index of 7 based on a range of 0–20. The Barthel Index reflects performance and mobility [32]. The participant had a Godin Leisure Time Physical Activity Scale [33] score of 3, indicating very low activity levels. The experimental procedure was approved by the University of Georgia Institutional Review Board and the participant gave written consent. All investigators involved in this study completed the CITI human subject certification.

### 4.2. Experimental Design

This was a case study involving a pre-post experimental design. Muscle endurance and mitochondrial capacity were measured before and after the training program. The training program took place over a 28-day period, with 12 sessions occurring within that time frame (3 days per week).

Training occurred in the Noninvasive Exercise Physiology lab on the University of Georgia campus. NMES was performed using 5 × 10 cm self-adhesive electrodes placed proximally and distally on the left brachioradialis muscle. NMES using biphasic 200 us pulses was performed using a muscle stimulator (Theratouch 4.7, Rich-Mar, Clayton, MO, USA). Stimulation intensity was set at 30 mA pulses based on the participant’s level of comfort and remained constant for all training sessions. Stimulation duration was 30 min. The first training session began at a stimulation frequency of 2 Hz. Stimulation frequency was gradually increased in subsequent training sessions based on feedback from the participants. The participant was encouraged to accept increased stimulation levels, and stimulation levels were adjusted both up and down during the training sessions based on directions from the participant.

Specific muscle endurance on the brachioradialis was measured in the left forearm of the participant [18]. While positioned supine, the participant laid with her forearm approximately 60° from the body and strapped down. The accelerometer (WAX3, Axivity Ltd., Newcastle upon Tyne, UK) is positioned in between the stimulation electrodes on the thickest part of the forearm. NMES was used to stimulate muscle contractions at 2 Hz, 4 Hz, and 6 Hz sequentially for 3 min each at 30 mA. A triaxial accelerometer was used to measure the magnitude of the resultant vector of each contraction. The endurance index for each frequency was the percentage of the acceleration at the end of each frequency period divided by the highest acceleration during 2 Hz [18].

mVO_2_max was measured in the brachioradialis muscle, as mentioned previously [18]. Near-infrared spectroscopy (Portamon, Artinis Medical Systems, Eisteinweg, The Netherlands) was used to measure oxygen saturation of hemoglobin/myoglobin in the muscle. The muscle metabolic rate was increased using NMES at 4 Hz for 30 s. NMES was followed by 18–22 short-duration cuff inflations, while muscle metabolism returned to a resting metabolic rate. Cuff inflation was performed using an occlusion cuff (Hokanson SC12D cuff, Bellevue, WA, USA) and a rapid cuff inflation system (Hokanson A10 and E20, Bellevue, WA, USA). Each occlusion produced linear decreases in oxygen levels in the muscle, the slope of which was the muscle metabolic rate (mVO_2_). The 18–22 mVO_2_ values were fitted to an exponential curve to calculate a rate constant. The rate constant was used as an index of mVO_2_max.

### 4.3. Analysis

Statistical evaluations were not performed in this case study. Differences between the pre-training and post-training conditions were compared to differences seen in training studies in other study populations.

## 5. Conclusions

Exercise is recommended for people with FRDA, and our study supports the use of NMES to enhance muscle function in people with FRDA. NMES has the potential to provide a wide range of benefits, including improving function in people with meaningful functional ability, improving the control of blood sugar, reducing the risks of developing vascular disease, and providing psychological benefits. The advantage of NMES is that it is not limited to people with the ability to perform significant voluntary exercise. This study demonstrated muscle improvements in a person with severe functional limitations. Future studies will be needed to evaluate NMES training for longer durations and apply NMES to people with a wider range of symptom severities. A better understanding of how people with FRDA respond to NMES training can help enhance function and potentially improve the quality of life for people living with FRDA.

## Data Availability

Data are available upon request.

## Supplementary Materials

The following supporting information can be downloaded at https://www.mdpi.com/article/10.3390/muscles4010001/s1. Data file: FA Summary Graph.
